# 

**Published:** 1884-06

**Authors:** 


### Contents—June.

ORIGINAL COMMUNICATIONS:

Fermentation in the Human Mouth. W. D. Miller .......... 281
Dental Education. B. Merrill Hopkinson .......... 291
Bromide of Ethyl as an Anæsthetic. G. L. Curtis .......... 297
Sputum-Septicæmia. W. D. Miller .......... 300
In Union, Strength. Henry N. Dodge .......... 301

REPORTS OF SOCIETY MEETINGS:

Mississippi Valley Dental Association .......... 302
American Medical Association .......... 309
New York Odontological Society .......... 313
Chicago Dental Society .......... 319
Southern Dental Association .......... 322

EDITORIAL:

An Item of Interest .......... 327
Legislation .......... 329
Dental Education .......... 331
Personal .......... 331
New York State Society Report .......... 332
Deferred .......... 332
Our Book Table .......... 332

CURRENT NEWS AND OPINION:

Western Miscellany .......... 335
What Does It Mean? .......... 336
A. D. S. E .......... 336
Missouri .......... 337
Sixth District Dental Society of New York .......... 337
Massachusetts Dental Society .......... 337
Change of Date .......... 337
Askings and Answers .......... 338

